# Continuous wave amplified spontaneous emission in phase-stable lead halide perovskites

**DOI:** 10.1038/s41467-019-08929-0

**Published:** 2019-02-28

**Authors:** Philipp Brenner, Ofer Bar-On, Marius Jakoby, Isabel Allegro, Bryce S. Richards, Ulrich W. Paetzold, Ian A. Howard, Jacob Scheuer, Uli Lemmer

**Affiliations:** 1 Light Technology Institute, Karlsruhe Institute of Technology, Kaiserstraße 12, 76131 Karlsruhe, Germany; 2 Department of Physical Electronics, Tel-Aviv University, Ramat-Aviv, 6997 Tel-Aviv, Israel; 3 Institute of Microstructure Technology, Karlsruhe Institute of Technology, Hermann-von-Helmholtz-Platz 1, 76344 Eggenstein-Leopoldshafen, Germany

## Abstract

Sustained stimulated emission under continuous-wave (CW) excitation is a prerequisite for new semiconductor materials being developed for laser gain media. Although hybrid organic-inorganic lead-halide perovskites have attracted much attention as optical gain media, the demonstration of room-temperature CW lasing has still not been realized. Here, we present a critical step towards this goal by demonstrating CW amplified spontaneous emission (ASE) in a phase-stable perovskite at temperatures up to 120 K. The phase-stable perovskite maintains its room-temperature phase while undergoing cryogenic cooling and can potentially support CW lasing also at higher temperatures. We find the threshold level for CW ASE to be 387 W cm^-2^ at 80 K. These results indicate that easily-fabricated single-phase perovskite thin films can sustain CW stimulated emission, potential at higher temperatures as well, by further optimization of the material quality in order to extend the carrier lifetimes.

## Abstract (web summary)

In order to develop perovskite-based lasers and LEDs for applications, their potential as room-temperature CW-pumped gain materials has to be established. Here, Brenner et al. demonstrate cw-pumped amplified spontaneous emission up to 120 K from a hybrid organic-inorganic halide perovskite layer.

## Introduction

Recently, solution-processed metal-halide perovskite semiconductors have started to challenge the performance of traditional inorganic semiconductors in the field of thin-film photovoltaics^1,2^. These perovskites possess the chemical structure ABX_3_, where A is an organic or inorganic cation, such as methylammonium (CH_3_NH_3_/MA), formamidinium (CH(CH_2_)_2_)/FA) or cesium (Cs), B is a metal (commonly Pb), and X is a halide (Cl, Br, or I). With an impressive tolerance to grain boundaries and defects, perovskite thin films exhibit high charge carrier mobilities, as well as excellent absorption and emission properties^3^. These properties of thin films of perovskite deposited, e.g., by spin-coating, can be surprisingly close to the performance of perovskite single crystals^4^. As a direct bandgap semiconductor, perovskites are well-suited not only for solar cells, but also for light-emitting devices^5,6^. Perovskites can be integrated in light-emitting diodes (LEDs) as the emitting semiconductor layer^6–9^, or applied in the form of nanocrystals for color conversion with InGaN-based LEDs as a potential replacement for traditional phosphors^10^. Of most relevance to the current work is the ability of perovskites to exhibit stimulated emission and serve as optical gain media in the near infrared and across the visible range^11,12^. The progress in perovskite lasers has been summarized in several recent reviews^6,13–18^. Low-threshold lasing under pulsed optical excitation has been demonstrated using various resonator geometries, including at wavelengths within the “green gap”^19,20^, where conventional III–V-semiconductors suffer from low efficiencies^21–23^. However, to become commercially relevant (ultimately in an electrically pumped configuration), any novel gain material (such as perovskites) should demonstrate the ability to maintain optical gain under continuous wave (CW) operation and ideally at room temperature, or at least at temperatures accessible by Peltier cooling (>220 K).

Obtaining CW operation has proven to be a major challenge for most solution-processable semiconductors, such as organic semiconductors and colloidal semiconductor nanocrystals. While the former class of materials has never demonstrated CW lasing, the latter only recently made this step, almost two decades after the initial investigations^24–27^. Despite this impressive achievement, further progress towards electrically pumped lasing is expected to be highly challenging for colloidal semiconductor nanocrystal devices due to their limited electrical transport characteristics.

Demonstrating CW lasing in thin perovskite films is of great interest, as this would present a solution-processable gain material exhibiting excellent electrical transport properties. However, to date CW stimulated emission has not been demonstrated in technologically relevant single-phase thin films. First demonstrations of CW lasing in perovskites were made in nanowires, where lasing was attributed to a condensate of polaritons enabled by the strong light–matter interaction between the photons and the cavity excitons^28,29^. True CW photon lasing in thin films, which is more relevant for practical applications, has proven to be difficult to achieve with the perovskite workhorse material methylammonium lead triiodide (CH_3_NH_3_PbI_3_). This difficulty has been ascribed to the phase instabilities of the crystal lattice with temperature, the limited thermal conductivity, screening effects and/or the rotational freedom of the methylammonium cation^30–32^. Clear proof of CW lasing in CH_3_NH_3_PbI_3_ was demonstrated by Jia et al. at a very specific temperature of 100 K, where different crystalline phases coexist upon optical pumping^32^. The requirement of the simultaneous presence of two crystal phases limited CW lasing to these special conditions, whereas at other temperatures, photon lasing has only been possible at pulsed excitation^32^. In this sense, although this previous demonstration of CW lasing was an impressive achievement, the need for the mixed-phase structure does not establish the ability of conventional, single-phase, perovskites to serve as gain material of sufficient quality for CW lasing.

Here, we demonstrate sustained CW lasing in a mixed cation system at the A site of the perovskite crystal structure. We use a mixed cation perovskite as it undergoes no phase change during the transition from room temperature to cryogenic temperatures. Consequently, we show that this phase-stable mixed-cationic perovskite can support CW optical gain at any temperature up to 120 K. These findings are a significant step towards practical perovskite CW lasers operating at room temperature, which opens new avenues to the development of perovskite semiconductor laser diodes and the solution of the green gap problem.

## Results

### Temperature-dependent amplified spontaneous emission (ASE) under pulsed excitation

In our study we employ a triple cation system with a non-stoichiometric ratio of Cs_0.1_(MA_0.17_FA_0.83_)_0.9_Pb_0.84_(I_0.84_Br_0.16_)_2.68_ in the precursor solution as reported previously in order to obtain low optical gain thresholds and an improved stability^12^. The partial replacement of the MA cation results in an identical crystal phase for arbitrary iodine bromine ratios at room temperature. No phase transitions are known at lower temperatures^33,34^, or have been observed in the current work. The perovskite layers were prepared on sapphire substrates and post treated by an imprinting step to reduce the surface roughness and to improve the morphology^35–37^ (see Supplementary Figure 1 for atomic force microscopy images of both pristine and imprinted layers). The smaller roughness decreases the waveguide scattering losses and is, therefore, expected to reduce the ASE threshold^12,35,38^. ASE, a type of mirrorless lasing^39^, is highly suited for studying the material’s gain characteristics by eliminating the influences of the laser resonator (which can induce a strong temperature dependence through the thermo-optic coefficient of the material). Additionally, this approach eliminates any ambiguity that may arise from cavity effects, such as angular-dependent diffraction of certain wavelengths from gratings or spectral filtering from DBR mirrors, which can change the detected shape of the spontaneous emission spectra even without stimulated emission being involved. Accurately establishing the existence of lasing is indeed not experimentally trivial and for more information on the experimental verification of real lasing the reader is referred to the commentary by Samuel et al.^40^.

**Fig. 1:**
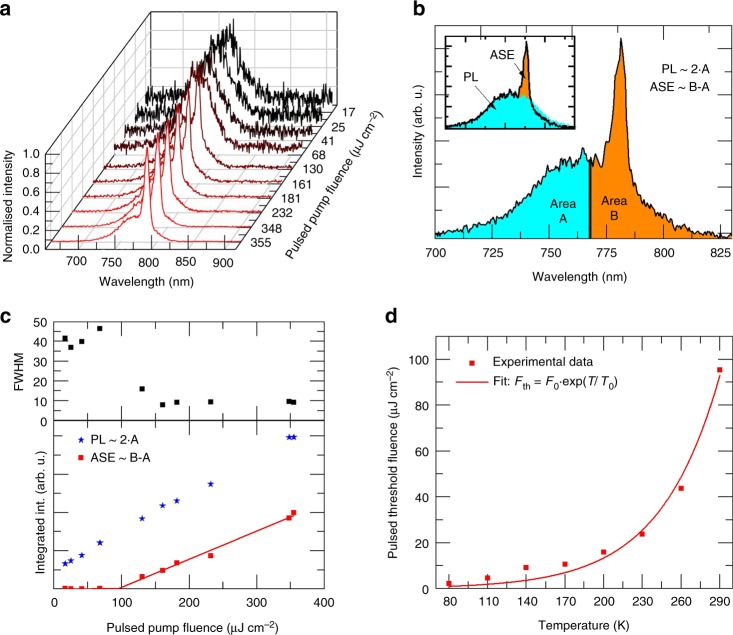
Amplified spontaneous emission properties under pulsed excitation (~0.8 ns). **a** Example of data for room temperature showing the evolution of the emission spectra from which the amplified spontaneous emission (ASE) threshold is established. Different colors for the spectra are chosen for better distinguishability. **b** Regions of the spectrum used to determine spontaneous emission (PL) and ASE contributions. The inset graphically shows the assigned PL contribution (blue, area A and area A mirrored at the center wavelength) and ASE contribution (orange, area B–area A) and the real spectrum (black line). **c** PL, ASE, and full width at half maximum (FWHM) as a function of the pulse fluence. **d** ASE threshold as a function of temperature. The threshold decreases by more than an order of magnitude from room temperature to 80 K. The red line is a fit to the empirical exponential threshold law typically observed in inorganic semiconductors. The characteristic temperature *T*_0_ is found to be 46 K

**Fig. 2:**
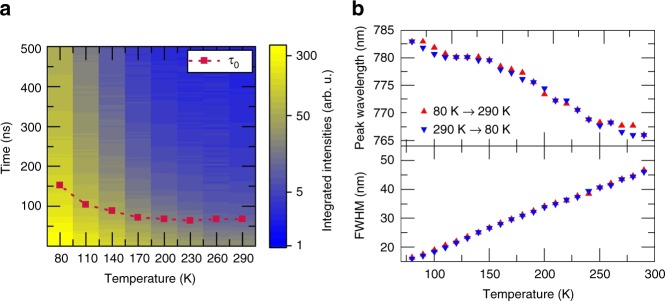
Temperature-dependent PL properties. **a** Transient PL images showing the integrated intensity as a function of time after fs-excitation and temperature at an excitation fluence of 60 nJ cm^−2^. The red dots denote the carrier lifetime *τ*_0_ defined as the time when the integrated intensity dropped to 1/e of its initial intensity. The dotted line is drawn as a guide to the eye. **b** Peak wavelength and FWHM of the thin film PL. The blue symbols are extracted from the measurement while cooling down and the red symbols are extracted while heating up

Figure 1 depicts the temperature-dependent ASE thresholds measured under 0.8 ns pulsed excitation. An example of the evolution of the emission spectra from a 185 nm-thick perovskite film over a range of pump fluences at room temperature is shown in Fig. 1a. The film was mounted inside a cryostat (Janis STVP-100, see Supplementary Figure 2 for a schematic of the experimental setup) allowing the intensity-dependence of the emission spectrum to be measured as a function of temperature. In order to unambiguously identify ASE, it is important to monitor the broadband spectra below threshold (the spontaneous emission (PL), PL refers to the spectral part not containing stimulated emitted photons), as well as the narrowband spectra above threshold. The change of the luminescence profile below and above threshold distinguishes the onset of ASE from artifacts in thin films, such as a spectral modification of the detected PL due to photon propagation in a waveguide close to cut-off conditions. At low excitation fluences, the PL spectrum can be clearly identified by a broadband emission profile with a full width at half maximum (FWHM) of ~40 nm and a nearly Gaussian shape. As the pump fluence increases, an intense emission shoulder between 773 and 785 nm (superimposed onto the PL spectra) emerges, indicating the onset of ASE. There are different methods to extract the threshold value for ASE^41^, here we follow the approach suggested by Sutherland et al.^42^. In the transition region from “predominant PL” to “predominant ASE”, the spectrum consists of a superposition of contributions from both processes. The PL contribution is proportional to the integral of the emission in the higher energy half of the PL-spectrum, which does not contain the ASE part (680–767 nm, area A in Fig. 1b). The ASE contribution can be estimated by integrating the low-energy half of the spectrum (area B in Fig. 1b) and subtracting the PL (area A) as illustrated in Fig. 1b. The integration boarder is chosen as the center wavelength of a Gaussian Fit to the spectra recorded at the lowest fluence (Supplementary Figure 3). Figure 1c depicts the total integrated PL intensity (approximated by twice the area A), the integrated ASE intensity (approximated by the difference between area B and area A) and the narrowing of the FWHM as a function of the pump fluence. The threshold level is determined by the intersection of a linear fit of the ASE contribution with the pump fluence axis, yielding a threshold level of 95.3 µJ cm^−2^. This value agrees well with the values reported in the literature for perovskite semiconductors^6,15,16^. Figure 1d depicts the dependence of the threshold levels on the temperature. As the temperature is reduced from 290 to 80 K, the ASE threshold decreases down to .2 µJ cm^−2^. It is empirically well-known that temperature-dependent current threshold in semiconductor diode lasers follows an exponential law^43^. Figure 1d shows a fit of the pulsed threshold fluence using the following formula:

1 $$F_{{\mathrm{th}}} = F_0 \cdot \exp \left( {\frac{T}{{T_0}}} \right)$$

where *T* is the temperature, *F*_0_ the threshold when *T* approaches zero and *T*_0_ is the characteristic temperature. As can be seen, there is a good agreement between the measurements and the fit, yielding a characteristic temperature of *T*_0_ = 46 K. We note that this characteristic temperature is lower than that of most inorganic semiconductor laser diodes, implying a stronger dependence of the threshold levels of our perovskites on the temperature^43^. The reduction of the thresholds at lower temperatures can be attributed mainly to two distinct mechanisms. First, the temperature dependence of the carrier distribution in the conduction and valence bands leads to lower threshold values for achieving population inversion at lower temperatures. However, this effect by itself yields a temperature dependence that scales as $T^{\frac{3}{2}}$ (see Supplementary Note 1), indicating the impact of additional mechanisms. Second, the recombination lifetime increases for lower temperatures, leading to larger carrier densities (at a given excitation intensity) at lower temperatures. Consequently, the threshold density level is obtained at lower pump fluences when the temperature is low. The dependence of the carrier lifetime on the temperature was carried out using streak camera measurements. Consistent with the literature^44^, a significant increase of the carrier lifetime was found when the temperature was reduced from 290 to 80 K (Fig. 2a and Supplementary Figure 4). Additionally, the total signal strength at constant excitation fluence increases substantially at lower temperatures due to the typical increase of the photoluminescence quantum yield (PLQY)^45,46^. Consequently, the ratio of radiative to non-radiative recombination increases, leading to a lower ASE onset threshold. In contrast to CH_3_NH_3_PbI_3_, where the structural phase change at 160 K leads to a drastic and sudden change in the PL properties, the PL spectrum in our triple cation material shifts steadily over the entire temperature range without any noticeable hysteresis (Supplementary Figure 5). The PL peak wavelength shifts gradually from 766 to 783 nm while its FWHM narrows from 46 to 16 nm as the temperature is decreased from 290 to 80 K (Fig. 2b), clearly indicating the existence of a single phase across the transition from room to cryogenic temperatures.

### ASE under CW excitation

**Fig. 3:**
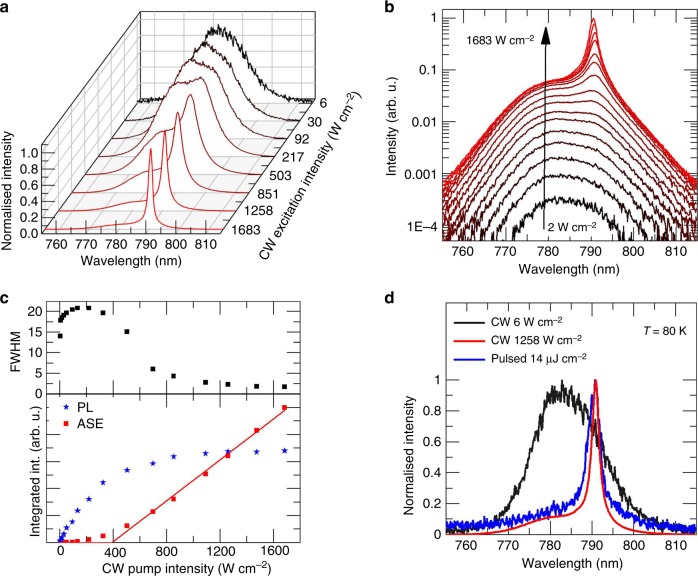
Characterization of CW ASE at 80 K. **a** The emission spectrum changes for increasing excitation intensities from a broadband PL spectrum (FWHM~15–20 nm) to a narrowband ASE spectrum (FWHM ~2 nm). **b** Logarithmic representation of the luminescence spectra for increasing excitation intensities showing the rise of ASE between 786 and 794 nm. **c** PL, ASE, and FWHM as a function of the CW excitation intensity. At the threshold intensity of 387 W cm^−2^, the FWHM decreases significantly and most of the additional pump intensity is fed into the ASE band. **d** Comparison of CW PL, CW ASE, and pulsed ASE spectra at 80 K

The source used for the CW pumping experiments was a diode-pumped solid-state laser (Millennia, Spectra Physics, 532 nm, *P*_max_ = 3.3 W). Figure 3a depicts the normalized emission spectra at several different CW pump intensities measured at 80 K. Similar to the pulsed excitation case, the typical ASE shoulder evolves in the spectra between 786 and 794 nm and develops into a dominating peak. Figure 3b depicts a log-scale plot of the luminescence spectra for all recorded pump intensities (Supplementary Figure 6 includes the detailed pump powers and include a linear scale plot, Supplementary Figures 7 and 8 depict the normalized spectra). Above the ASE threshold, most of the additional pump power is fed into ASE when the excitation intensity is increased. Using the above-mentioned approach, the ASE threshold intensity under CW excitation was found to be *I*_th,CW_ = 387 W cm^−2^ (see Fig. 3c). Note that above threshold, the FWHM of the ASE peak collapses rapidly, reaching values of ~2 nm at high pumping intensities. The clear threshold and the dramatic narrowing of the peak proves the generation of ASE under CW excitation. Figure 2d presents a comparison of the emission spectra under CW excitation at low intensity (well below threshold, 6 W cm^−2^), at high intensity (well above threshold, 1258 W cm^−2^), as well as the corresponding spectra under pulsed excitation above threshold fluence (~14 µJ cm^−2^). The spectra at high CW intensities and pulsed excitation are comparable, proving that the material’s emission properties and the crystal phase remain unchanged when CW ASE starts. Note that sustaining the same crystal morphology under pulsed and CW excitation constitutes a significant improvement with respect to the previous CW lasing observations in CH_3_NH_3_PbI_3_^32^, thus demonstrating that CW stimulated emission can be sustained in single-phase perovskites. The limited thermal conductivity of perovskites does not seem to be a major issue since the ASE peak wavelengths for pulsed and CW excitations are identical.

The CW threshold *I*_th,CW_ value is nearly an order of magnitude lower than the corresponding peak intensity at pulsed excitation (*I*_th,pulsed_ = 2750 W cm^−2^), calculated as the threshold fluence divided by the pump duration (0.8 ns) under pulsed excitation. This is, however, not surprising as the carrier lifetime at 80 K is substantially longer than the pump pulse duration. When the excitation pulse is shorter than the carrier lifetime, the threshold power and the pulse energy are rather related by^47^

2 $$I_{{\mathrm{th}},{\mathrm{CW}}} \cdot \tau = F_{{\mathrm{th}}}$$

where *τ* is the carrier lifetime. Dividing our observed values of *F*_th_ and *I*_th,CW_ results in a coarse estimate for the carrier lifetime at low temperature of 5.7 ns, which is entirely reasonable considering our streak camera measurements, even when considering the significant additional Auger processes for higher excitation densities (Fig. 2a).

The plausibility of the ASE threshold can be tested using the Bernard–Duraffourg condition^48^ which, for equal effective masses for both carriers, requires the quasi-Fermi levels for electrons and holes to reach the conduction and valence band energy levels, respectively. Assuming *m*_e_ = *m*_h_ for perovskite semiconductors^3^ and using standard equations for the carrier density in 3D semiconductors with parabolic bands, the carrier density at the point when the quasi-Fermi levels reach the band edges can be estimated to be 1.54 × 10^17^ cm^−3^ at 80 K (see Supplementary Note 1 for detailed calculations). Therefore, the minimal carrier lifetime required for reaching a given carrier density at a given excitation rate can be readily estimated (see Supplementary Note 2 for details). Using a generation rate based on the threshold intensity of 387 W cm^−2^, we find that a carrier lifetime of at least 3.9 ns is required in order to reach (or exceed) a density of 1.54 × 10^17^ cm^−3^_,_ required for net optical gain. As seen in Fig. 2a, the measured carrier lifetime at low excitations is well above this value.

**Fig. 4:**
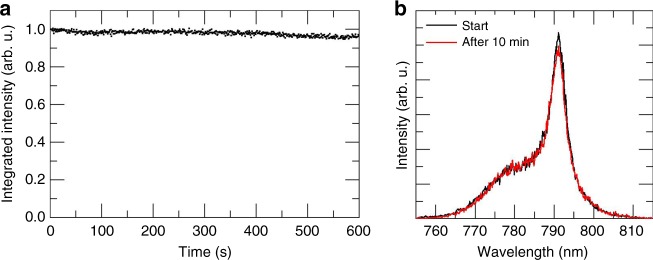
ASE stability at CW excitation with an excitation intensity of 720 W cm^−2^ at 80 K. **a** Integrated emission intensity over a time frame of 10 min of continuous excitation. **b** Spectra at the beginning of the measurement (black) and after 10 min (red)

In addition to low thresholds, lasing stability is an important requirement for any laser gain medium. Figure 4a shows the integrated emission intensity under constant CW excitation of 720 W cm^−2^ for a period of 10 min at a temperature of 80 K. Figure 4b depicts the corresponding spectra at the beginning and at the end of the measurement. A small decrease of the integrated and peak intensities of the ASE is visible, nevertheless, the stability of the emission is remarkable at such harsh excitation conditions. This demonstrates that the stability under CW excitation is comparable to the stability under pulsed excitation reported previously^11,12,32,49^. Additionally, we note that the wavelength of the ASE peak did not change during the experiment. The absence of such a shift, which is observed in CH_3_NH_3_PbX_3_ perovskites due to a redistribution of the halides^50^, confirms the stability of the perovskite crystal lattice with mixed cations at the A-site^33,34^.

**Fig. 5:**
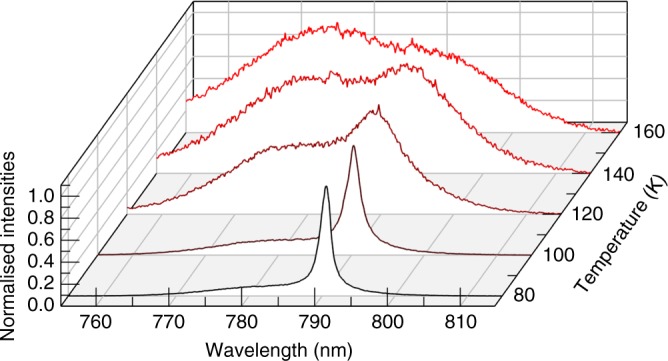
ASE spectra under CW excitation at various temperatures and constant pump intensity. The amount of photons emitted into the ASE mode decreases with increasing temperature until ~120 K, after which spontaneous emission dominates. The excitation power was kept constant at 1512 W cm^−2^

Next, we tracked the emission properties at higher temperatures under fixed CW pumping conditions as shown in Fig. 5. All measurements were carried out at a CW pump intensity of 1512 W cm^−2^. As the temperature is increased, the ASE peak decreases and a larger portion of the absorbed photons is reemitted by spontaneous emission (instead of stimulated emission). The ASE peak fades out at temperatures above 120 K. At this temperature (and above), a further increase of the pump intensity could not recover the ASE peak, but rather led to a fast degradation of the PL intensity. This indicates that at higher temperatures the material degrades before the CW ASE threshold is reached. However, the fact that the ASE peaks can be observed over the full range between 80 and 120 K, is another indication that the ASE is obtained by a single crystalline phase and not due to the coexistence of different phases as in CH_3_NH_3_PbI_3_, which requires a very specific temperature^32^.

Several approaches can be employed in order to increase the temperature range where ASE can be achieved before material degradation starts. This includes better thermal management, such as pumping the layer using photons with lower energy, closer to the bandgap, consequently introducing less heating during the thermalization of carriers to the band edges. In addition, an enhancement of the carrier lifetime and the PLQY at higher temperatures by a further improvement of the material quality should also allow for ASE generation at higher temperatures. Furthermore, the use of high-Q cavities can decrease the threshold values for laser devices compared to our ASE threshold in thin films. Our work manifests a crucial step towards the realization of a new class of laser materials, which can potentially support room-temperature CW lasing under optical pumping. In view of the high charge carrier mobilities in our material, this also raises prospects for future electrically pumped devices. Thus, a new class of low-cost and solution processable laser diodes with tunability over the full visible spectrum might be at the horizon.

## Methods

### Materials and sample preparation

The precursor powders PbI_2_ (AlfaAesar), PbBr_2_ (AlfaAesar), CsI (Alfa Aesar), FAI (Greatcell Solar Ltd.), and MABr (Greatcell Solar Ltd.) were mixed in a non-stoichiometric ratio of Cs_0.1_(MA_0.17_FA_0.83_)_0.9_Pb_0.84_(I_0.84_Br_0.16_)_2.68_ and dissolved in a mixture of dimethylformamide (DMF, Sigma Aldrich) and dimethyl sulfoxide (DMSO, Sigma Aldrich) in a ratio of 3:1. All chemicals and solvents were used as received without further purification. The sapphire substrates (1‶ diameter, 1 mm thick, Plano GmbH) were cleaned in an ultrasonic bath using acetone and isopropanol for 10 min each and treated by O_2_ plasma for 2 min at 100 W. Directly after the plasma treatment 70 µl of the solution were dynamically spin-casted at 1000 rpm for 10 s and 6000 rpm for 20 s in nitrogen atmosphere. Six seconds before the end of the spinning process, 160 μl of chlorobenzene were dropped onto the spinning sample. The resulting films were annealed at 100 °C for 60 min in N_2_. Afterwards, the films were post treated by imprint lithography. The imprint step used in this work is based on previously published studies^35–37^ and conducted using a commercially available nanoimprint lithography instrument (FC-150, SET) with the following recipe: A glass substrate covered with a thin layer of OrmoStamp^®^ was pressed into the perovskite substrate with a pressure of 10 bar to form an initial contact between the mold and the substrate. This was followed by heating the system to 125 °C, the final imprint temperature. Then, the imprint pressure was raised to 125 bar for a duration of 6 min followed by the cooling of the system for a duration of 6.5 min to achieve a final temperature of ~50 °C.

### ASE measurements

A schematic of the gain characterization setup is shown in Supplementary Figure S2: For the experiments under pulsed excitation, a frequency doubled Nd:YLF laser (Innolas GmbH), emitting pulses of 0.8 ns pulse duration at 532 nm was used. For the CW excitation experiments, a CW laser emitting at the same wavelength was used (Millennia, Spectra Physics). The pump intensities were controlled using variable neutral density filter wheels. The laser power was measured using a Coherent LabMax2 power meter with various power heads. The samples were mounted in an optical cryostat (Janis STVP-100) and connected via heat-conductive paste to a copper sample holder. The spot area at the sample position inside the cryostat was determined by the moving knife edge method to be 1.28 × 10^−4^ cm^2^ for the pulsed beam and 5.01 × 10^−4^ cm^2^ for the CW laser beam. The sample emission was coupled through a pair of lenses to a spectrograph (Acton Research Corporation, SpectraPro 300i, variable grating) connected to an intensified CCD camera (Princeton Research, PiMax 512).

### Time resolved and temperature-dependent PL

The PL transients in Fig. 2a were recorded by a streak camera (Hamamatsu Universal C10910) coupled to a spectrometer (Acton SpectraPro SP2300). The camera was used in single sweep mode to allow for a 2 μs time window and a FWHM of the instrumental response function of 28 ns. The steady-state PL spectra shown in Fig. 2b were recorded by a fiber-coupled spectrometer (Avantes, AvaSpec-2048L). In both cases, the temperature of the sample was controlled by a closed cycle cryostat (Oxford, Optistat Dry TLEX). For the excitation, a second harmonic generator (Coherent, Chameleon Compact OPO-Vis) was used to double the frequency of a mode-locked Ti:sapphire laser (Coherent, Chameleon Ultra) with a wavelength of 960 nm, a pulse width of 140 fs and a repetition rate of 80 MHz. The repetition rate of the laser system was reduced to 400 kHz using a pulse picker (APE, pulseSelect).

## Supplementary information

Supplementary Information

## Data Availability

The data that support the findings of this study are available from the corresponding author upon reasonable request.
