# Supplementary material for: Continuous wave amplified spontaneous emission in phase-stable lead halide perovskites
Source: Nat Commun. 2019 Feb 28;10:988. doi: 10.1038/s41467-019-08929-0 (PMC6395683; doi:10.1038/s41467-019-08929-0)
Supplement: Supplementary file 1 — Supplementary Information [file 41467_2019_8929_MOESM1_ESM.pdf]

Supplementary Information for

Continuous Wave Amplified Spontaneous Emission in Phase-Stable Lead Halide Perovskites

Philipp Brenner, Ofer Bar-On, Marius Jakoby, Isabel Allegro, Bryce S. Richards, Ulrich W. Paetzold, Ian A. Howard, Jacob Scheuer and Uli Lemmer

Supplementary Note 1:

Estimation of carrier density necessary for stimulated emission (Bernard-Duraffourg condition):

A prerequisite for gain in semiconductors is the Bernard-Duraffourg condition, meaning that the separation of the quasi-Fermi levels needs to exceed the photon energy[1]:

$$F_{\mathrm{C}} - F_{\mathrm{V}} \geq E_{\mathrm{g}} \geq h\nu \quad (1)$$

where F_{C} and F_{V} are the quasi-Fermi levels of the conduction and the valence band, respectively.

In quasi-equilibrium (after intraband relaxation, typically happening on a time scale shorter than a few ps)[2], a general relation between the carrier densities n or p and the quasi-Fermi levels $E_{\mathrm{F_C}}$ or $E_{\mathrm{F_V}}$ is given in a 3-D semiconductor by the following expression[3]:

$$n = N_{\mathrm{C}}(T) \cdot F_{1/2}\left(\frac{E_{\mathrm{F_C}} - E_{\mathrm{C}}}{k_{\mathrm{B}}T}\right) \quad (2)$$

$$p = N_{\mathrm{V}}(T) \cdot F_{1/2}\left(\frac{E_{\mathrm{V}} - E_{\mathrm{F_V}}}{k_{\mathrm{B}}T}\right) \quad (3)$$

where N_C and N_V are the effective densities of states for the conduction and valences bands, E_C and E_V are the conduction and valence band edge energies and $F_{1/2}(\xi)$ is the Fermi-Dirac Integral of order 1/2. The effective densities of states in a 3-D semiconductors are given by:

$$N_C(T) = 2 \cdot \left(\frac{2\pi m_{\mathrm{e}}^* k_{\mathrm{B}}T}{h^2}\right)^{3/2} \quad (4)$$

$$N_V(T) = 2 \cdot \left(\frac{2\pi m_{\mathrm{h}}^* k_{\mathrm{B}}T}{h^2}\right)^{3/2} \quad (5)$$

Assuming the same effective mass for electrons and holes which is a valid approximation according to literature[4], and taking a value of $0.15m_{\mathrm{e}}$, which was calculated and measured for perovskites near the band edge[4,5], the Bernand-Durrafourg condition is reached as soon as the quasi-Fermi levels reach the

values of the conduction and valence band edge energies. This means that $E_{F_C} = E_C$ and $E_V = E_{F_V}$ and it follows that the minimal needed carrier density at room temperature (293 K) is:

$$n_{\text{Threshold}} = N_C(293K) \cdot F_{1/2}(0) \approx 1.08 \cdot 10^{24} m^{-3} = 1.08 \cdot 10^{18} \text{cm}^{-3} \quad (6)$$

And for 80 K the thresholds carrier density results in:

$$n_{\text{Threshold}} = N_C(80K) \cdot F_{1/2}(0) \approx 1.54 \cdot 10^{23} m^{-3} = 1.54 \cdot 10^{17} \text{cm}^{-3} \quad (7)$$

Supplementary Note 2:

Minimal required carrier lifetime for a given carrier density:

A specific carrier density needed to obtain stimulated emission must be supplied by optical pumping. For CW pumping, a lower bound of the time t needed to accumulate this amount of charges can be approximated (neglecting all additional losses or recombination during that time (radiative or nonradiative)) by the following formula:

$$t = \frac{n_{\text{threshold}}}{\text{carrier generation rate}} \quad (8)$$

$$\text{carrier generation rate} = \frac{I_{\text{pump}}(1-R)}{\hbar \cdot \omega \cdot d} \cdot (1 - \exp(-\alpha \cdot d)) \quad (9)$$

With the experimentally determined CW threshold of $I_{\text{pump}} = 387 \text{ W cm}^{-2}$ at 80 K, at a pump wavelength of $\lambda_{\text{pump}} = 532\,\text{nm}$ and a film thickness $d = 185\,\text{nm}$, an absorption coefficient of $10^5\,\text{cm}^{-1}$ and a refractive index of 2.4, this lower bound can be calculated to be $t = 3.9\,\text{ns}$. Since the true time until stimulated emission can start after pump turn on will be longer due to carrier recombination, the carrier lifetime should not be significantly smaller, otherwise it would be impossible to accumulate this carrier density by CW pumping at the corresponding intensity.

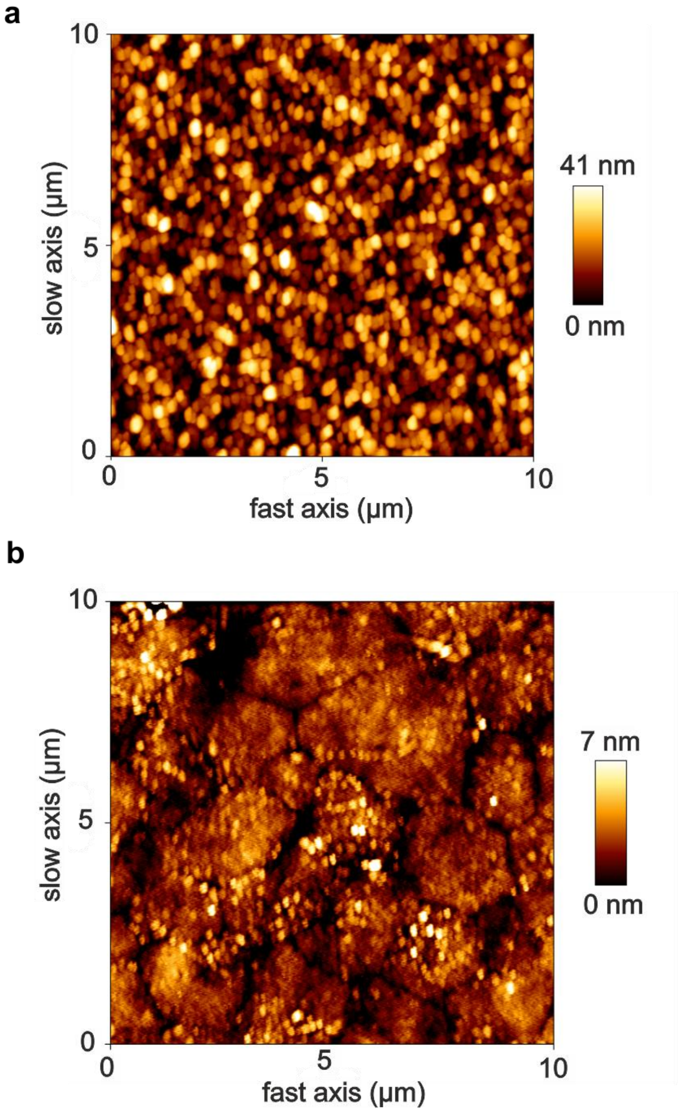

Supplementary Figure 1. AFM images of perovskite layers. a, Outside the imprint area. **b,** Inside the imprint area (where the measurements were taken). Note the different scales in the images. The perovskites grains are significantly enlarged by the imprinting process and the RMS roughness reduces from 9.2 nm to 1.4 nm.

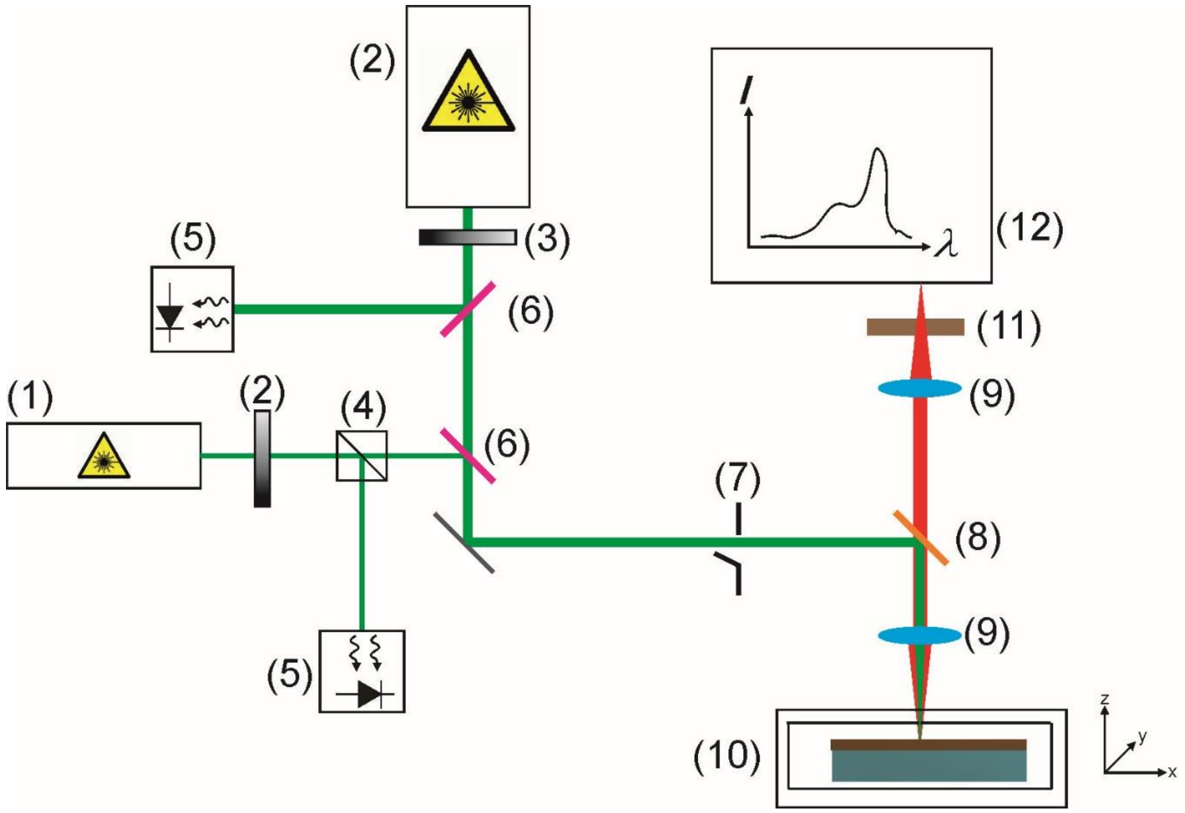

Supplementary Figure 2. Schematic of the optical characterization setup. (1): Pulsed laser (Piccolo Mopa Innolas, 532 nm, 0.8 ns). (2): Continuous wave laser (Millenia, Spectra Physics 532 nm). (3): Variable neutral density filter. (4): Beam splitter. (5): Energy reference. (6): Flip mirror. (7) Mechanical Shutter. (8): Dicroic Mirror. (9): Lens (10): Optical Cryostat (Janis STVP-100). (11): Long Pass (630 nm). (12): Spectrograph (SpectraPro 300i, Acton) with an iCCD Camera (Princeton Research, PiMax 512).

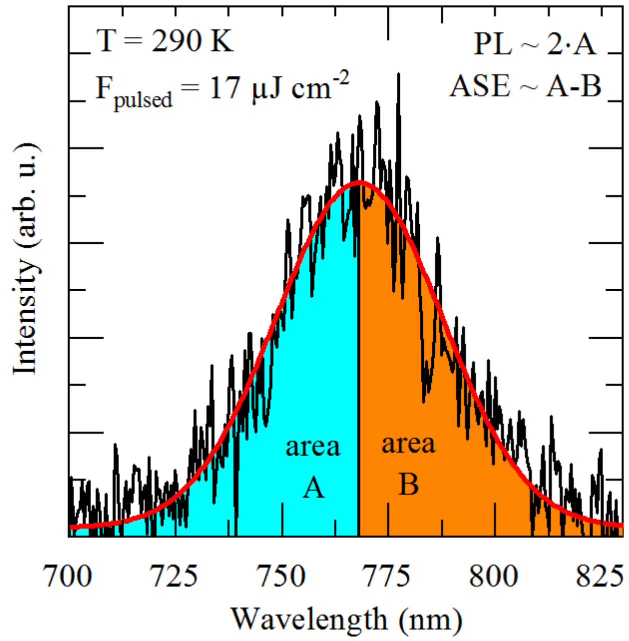

Supplementary Figure 3. PL spectrum at 290 K under pulsed excitation used for determining the PL and ASE contributions. The integration boarder for determining the PL (~2·A) and ASE contribution (~B-A) are selected as the center of a Gaussian fit to the PL spectrum at the lowest recorded fluence (17 μJ cm^{-2}) for each temperature.

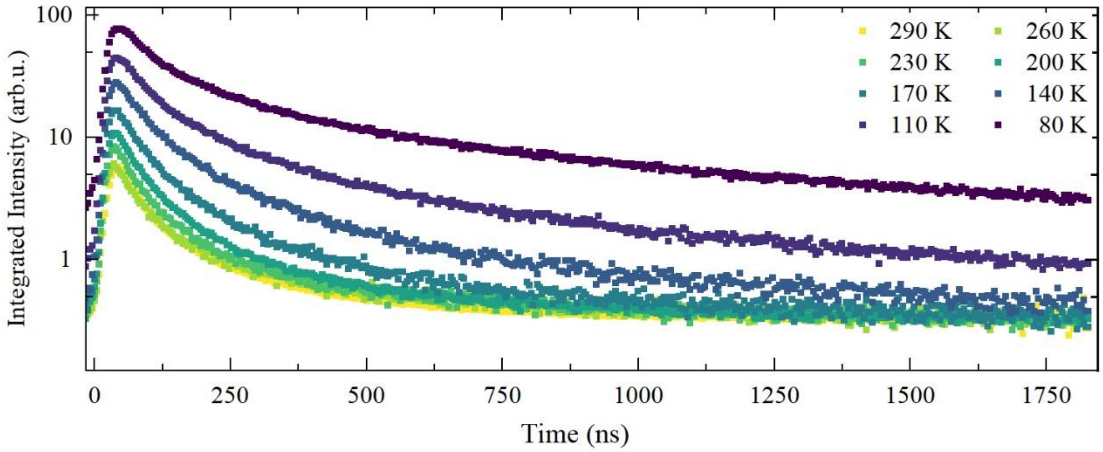

Supplementary Figure 4. Transient photoluminescence intensities. Recorded at a constant fluence of 60 nJ cm^{-2}. With decreasing temperature, the intensity and the carrier lifetime increases.

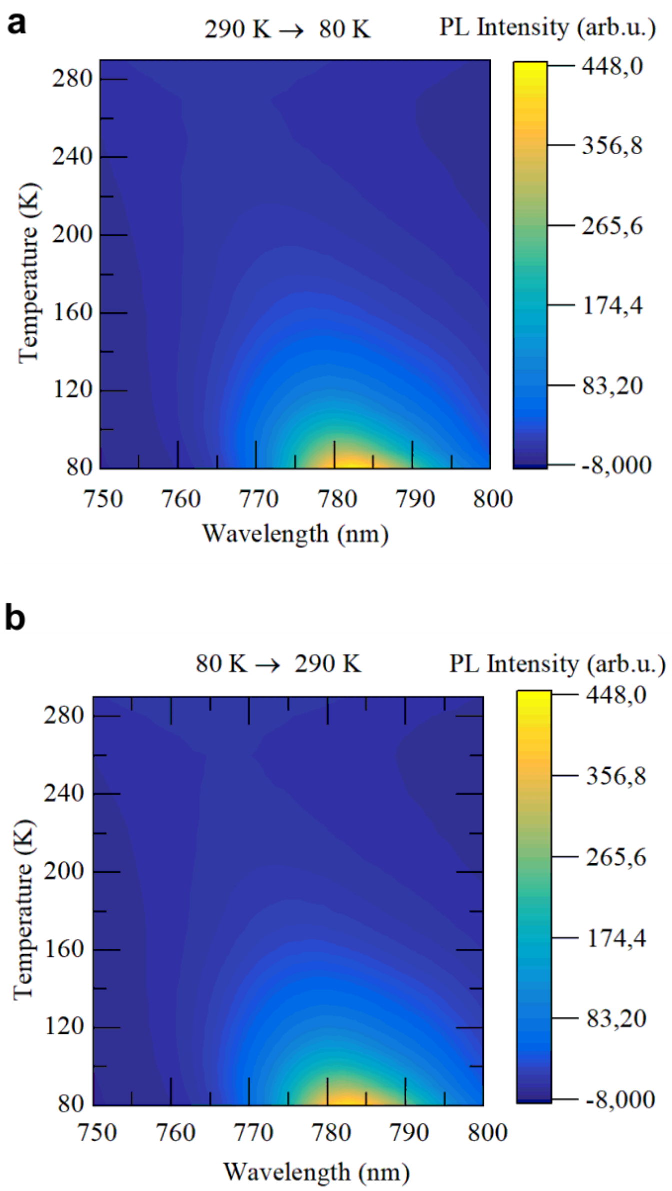

Supplementary Figure 5. Temperature dependent PL maps. a, PL intensity while cooling from 290 K to 80 K. **b,** PL intensity while heating from 80 K to 290 K. The spectral development has no discontinuities and no hysteresis, which indicates that the crystal phase stays the same over the investigated temperature range.

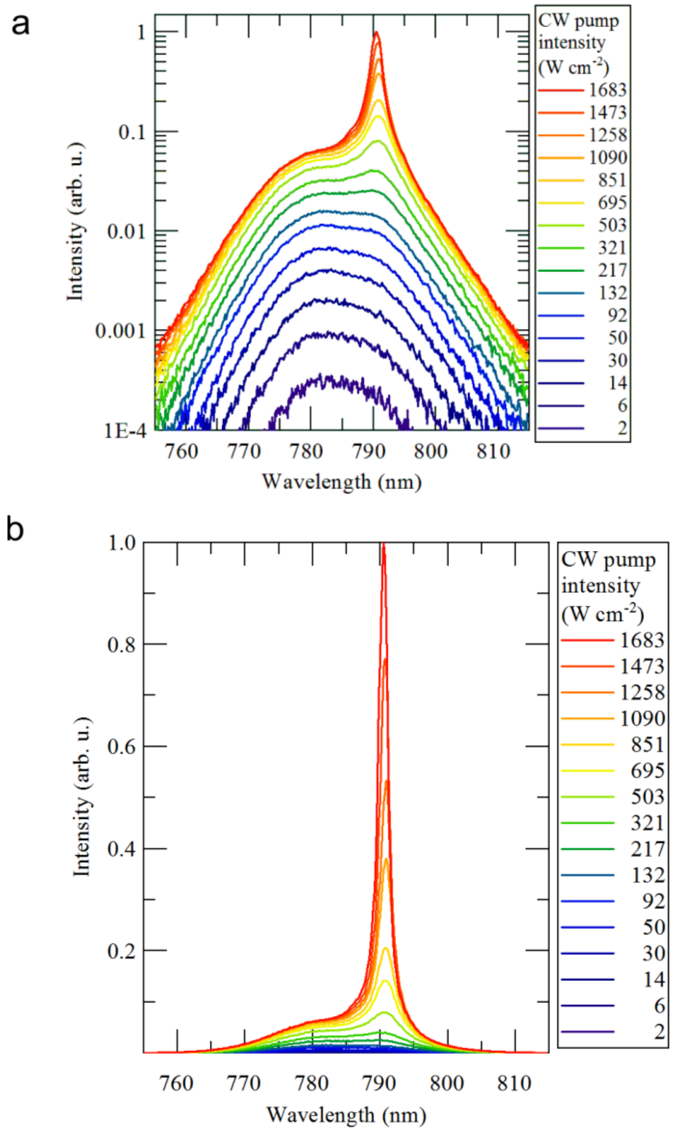

Supplementary Figure 6. Spectra under CW excitation. a, on a log-scale and **b,** on a linear intensity axis. Same data as in Fig. 3b. It can be seen, that the narrowband ASE dominates over PL. On a linear axis, however, PL is difficult to see due to the low intensity.

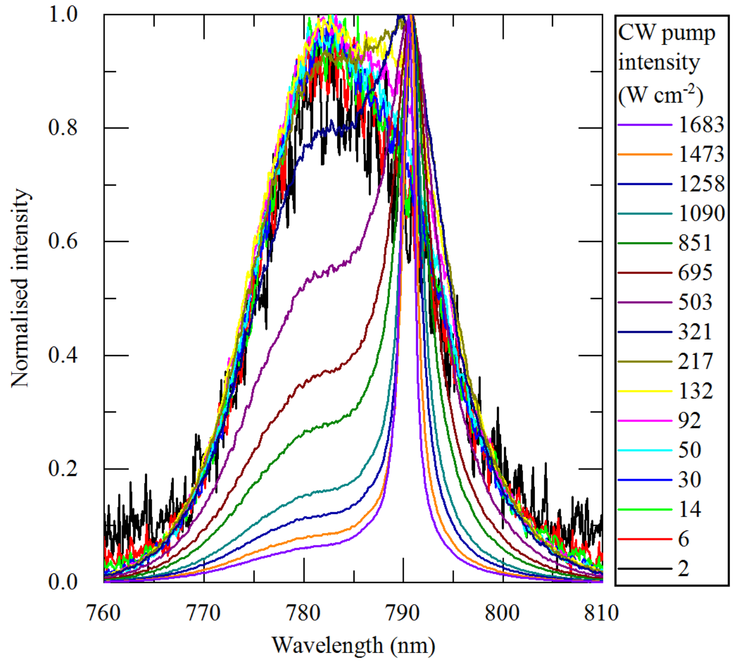

Supplementary Figure 7. Normalized spectra under CW excitation. With and individual normalization of each spectra, it can be clearly seen when the spectra starts to change from a broad PL at low intensities to a narrow ASE spectrum at high intensities.

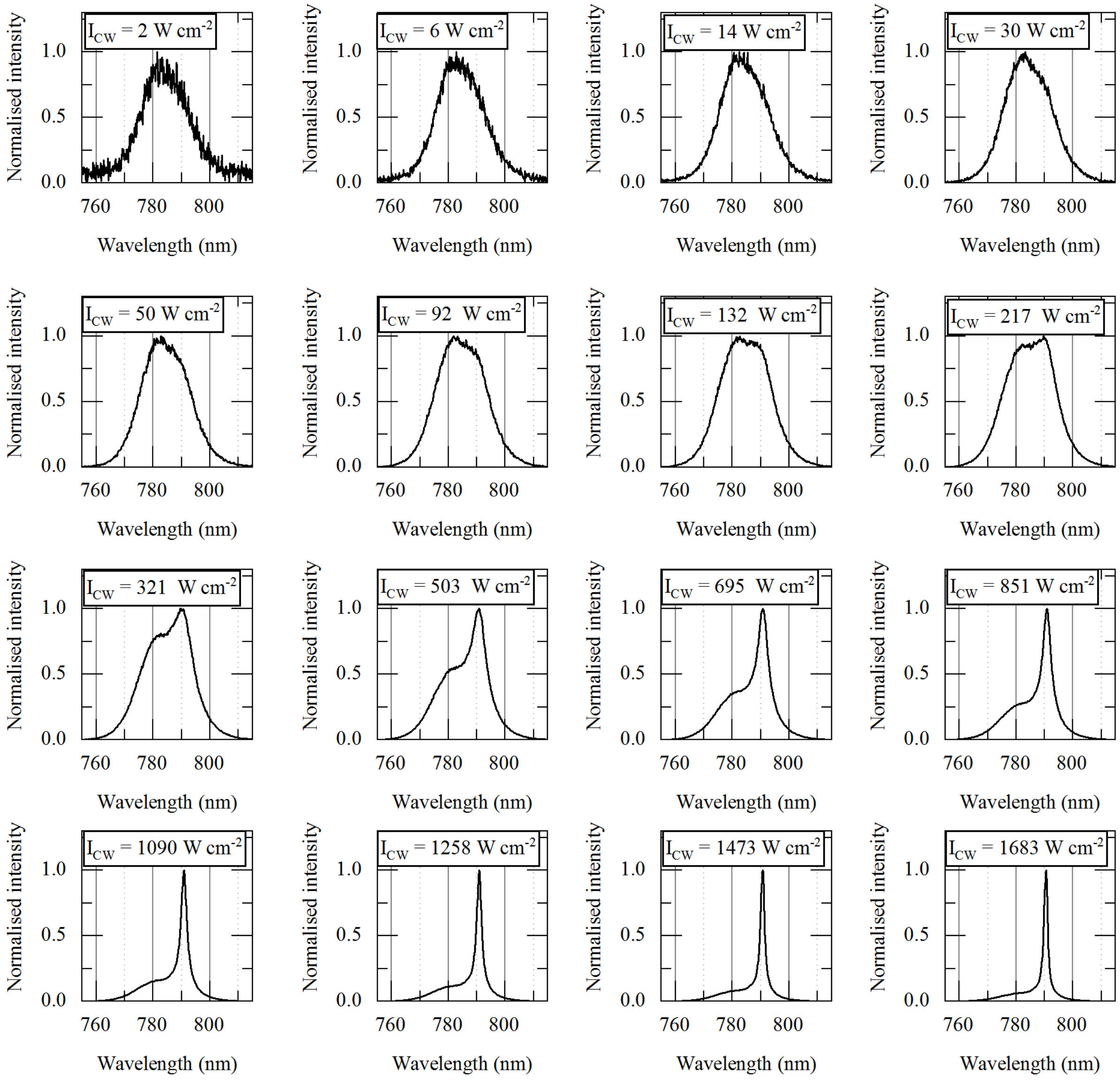

Supplementary Figure 8. Individual spectra under CW excitation at 80 K.

Supplementary References:

1. Bernard, M. G. A. & Duraffourg, G. Laser Conditions in Semiconductors. *Phys. Status Solidi* **1,** 699–703 (1961).
2. Richter, J. M. *et al.* Ultrafast carrier thermalization in lead iodide perovskite probed with two-dimensional electronic spectroscopy. *Nat. Commun.* **8,** 1–7 (2017).
3. Liu, J. M. *Photonic Devices*. (Cambridge University Press, 2005).
4. Herz, L. M. Charge-Carrier Mobilities in Metal Halide Perovskites: Fundamental Mechanisms and Limits. *ACS Energy Lett.* **2,** 1539–1548 (2017).
5. Li, G., Price, M. & Deschler, F. Research Update: Challenges for high-efficiency hybrid lead-halide perovskite LEDs and the path towards electrically pumped lasing. *APL Mater.* **4,** 091507 (2016).
